# Microfluidic Systems for Pathogen Sensing: A Review

**DOI:** 10.3390/s90604804

**Published:** 2009-06-17

**Authors:** Jürgen Mairhofer, Kriemhilt Roppert, Peter Ertl

**Affiliations:** 1 Department of Biotechnology, University of Natural Resources and Applied Life Sciences, Muthgasse 18, 1190 Vienna, Austria; 2 Division of Nano-System-Technologies, Austrian Research Centers GmbH – ARC, Donau-City-Street 1, 1220 Vienna, Austria

**Keywords:** microfluidics, pathogen sensing, lab-on-a-chip

## Abstract

Rapid pathogen sensing remains a pressing issue today since conventional identification methodsare tedious, cost intensive and time consuming, typically requiring from 48 to 72 h. In turn, chip based technologies, such as microarrays and microfluidic biochips, offer real alternatives capable of filling this technological gap. In particular microfluidic biochips make the development of fast, sensitive and portable diagnostic tools possible, thus promising rapid and accurate detection of a variety of pathogens. This paper will provide a broad overview of the novel achievements in the field of pathogen sensing by focusing on methods and devices that compliment microfluidics.

## 1. Introduction

**Figure 1.**
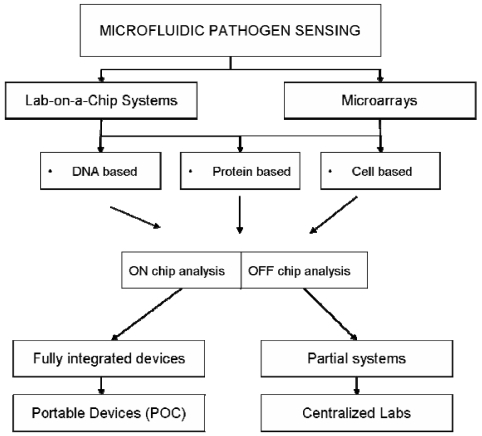
Schematic overview of microfluidic based pathogen sensing systems.

The rapid detection and identification of microorganisms is a pressing issue in fields ranging from clinical diagnostics and monitoring of food-borne pathogens to detection of biological warfare agents. It is well known that health care systems would greatly benefit from faster, more accurate diagnosis to significantly reduce health care costs, while simultaneously providing better epidemiological data that can be used for infectious disease modeling [1]. Despite substantial progress made in the diagnostic field, there is still a need for faster, portable and more accurate diagnostic methods. The ongoing search for improved methodologies is particularly important since conventional identification methods of pathogenic microorganisms usually require large cell numbers of a pure cell culture, involving time and labor consuming enrichment and pre-selection steps. For instance, the developed world standards for target pathogen diagnosis, including culture, enzyme immunoassay and polymerase chain reaction (PCR), often take between 2 to 4 days. Furthermore, since most centralized laboratories are limited to large cities, near-patient testing using point of care (POC) devices has become increasingly important. Therefore, robust and portable diagnostic devices capable of rapidly providing information on pathogens will also help reduce mortality rates, hospitalization and timely isolation in case of infectious pathogens. Although, a variety of different biosensors have been developed in the past two decades, there is still a need for miniaturized, low-cost or disposable biosensors capable of rapid detection and accurate identification of a wide range of pathogens. Recent efforts to minimize the time span between sampling and results include the use of miniaturized devices that do not depend on special infrastructure and sample preparation procedures [2]. The area of miniaturized or microfluidic analysis systems, also called “micro total analysis systems (μTAS) or lab-on-a-chip (LOC)”, has gained increased popularity [3]. Initially, the main reason for miniaturization was to enhance analytical performance, but the reduction of size also presented the advantages of reduced consumption of reagents and the ability to integrate separation and monitoring techniques within a single device [4]. The ability of microfluidic systems to conduct measurements from small volumes of complex fluids with efficiency and speed, without the need for a skilled operator, has been regarded as the most powerful application of Lab-on-a-Chip (LOC) technologies [1]. Furthermore, portable LOC devices capable of automated complex diagnostic procedures, normally performed in a centralized laboratory, are able to provide healthcare workers and outpatients with important health-related information even in the most remote settings. Portable medical diagnostic tools are of great importance in developing countries [5] where more than half the deaths are attributed to infectious diseases [6]. Overall, the interest of moving to a more patient-centric and home-testing approach is on the rise and microfluidic lab-on-a-chip technology suits both developing and developed-world applications. In this paper, the recent progress within microfluidics based pathogen sensing is reviewed. We have organized this review into various sections addressing the various methods used for microchip fabrication, pathogen detection and commercial applications. Additionally, within the pathogen detection section, an overview of the microfluidic based pathogen detection methods is provided according to target analytes such as DNA, proteins and whole cells. Figure 1 presents a schematic overview of the field of microfluidic based pathogen sensing.

## 2. Materials, Manufacturing and Detection Methods of LOC Devices

Most of the manufacturing methods used for microfluidic biochips were developed in the semiconductor industry [7]. Consequently, a feature common to biosensors, microfluidics and biochips is that photo-lithographic processes are employed in their fabrication and substrates such as silicon, glass or quartz are used [8]. The greatest benefit of chip technology is miniaturization because it offers innovative capabilities and improved performance over current technologies. For example, the manipulation of nanoliter to picoliter volumes on silicon chip surfaces has led to chemical microreactors and enhanced detection limits [9,10]. Additionally, improved performance is also a fundamental component for the development of high-sensitivity, real-time cellular analysis technologies [11,12]. Over the years a variety of materials have been used for microfabrication including silicon, glass, soft or hard polymers, as well as biomaterials such as calcium alginate and cross-linked gelatin or hydrogels [13]. However, a recent trend moving towards polymer microfabrication technologies is observed in the literature, due to efforts to minimize the cost of the microfluidic devices [14]. This is also true in the field of pathogen sensing, where most applications demand disposable systems to eliminate the risk of cross-contamination. In general, polymeric materials of choice can range from solvent resistant materials such as Teflon^®^, photopatternable silicon elastomers, thermoset polysters, poly(methylmethacrylate) (PMMA) and patterned poly-(dimethylsiloxane) (PDMS), polyimide and SU-8 (negative photoresist) polymers [15-18]. Challenges facing plastic based microfluidic devices include minimization of batch-to-batch variations, improvement in chemical resistance, control over surface chemistry and compatibility with fluorescence [8]. It is also important to note that a variety of operations need to be performed with LOC devices during operation, such as sample pre-treatment adapted to the source of physiological fluids (e.g. blood, saliva and urine), fluid actuation (e.g. passive or active) and control (e.g. mixing) as well as signal detection. Additionally, there are also specific transportation issues in a variety of environments that need to be considered such as temperature changes and high humidity [19].

Virtually all analytical detection methods have been successfully integrated or coupled with LOC devices, including optical detectors, electrochemical detectors, magneto-resistive sensors (GMR), acoustic and mass spectrometric (MS) as well as nuclear magnetic resonance (NMR) ones, respectively [20-24]. However, optical and electrochemical sensors are probably the most popular in pathogen analysis due to their selectivity and sensitivity [25-29]. In general it is convenient to incorporate conventional optical or electrochemical devices with microfluidic detection systems [30-33]. For instance, the application of photo-diodes allows for the integration of optical detectors with microfluidics to make portable lab-on-a-chip systems. For instance, a microfluidic ATP-bioluminescence sensor for the detection of airborne microbes using commercial available photo-diodes has been recently reported [27]. Although optical absorption detection is compatible with microfluidics, they suffer from relatively poor detection limits due to the short effective path length found in microfluidic channels [34]. Consequently, fluorescence detection remains the dominant optical detection technique in microfluidics. Here the conjugation of affinity markers (e.g. antibodies, DNA etc.) with fluorescent compounds like fluorescein isothiocyanate (FITC), phycoerythrin (PE) cyanin- or Alexa-dyes is most commonly used. Alternative approaches are based on the incorporation of two fluorescence molecules into the biosensor, using fluorescence resonance energy transfer (FRET) [35]. Other optical methods include chemiluminescence (CL), bioluminescence (BL) and Surface Plasmon Resonance (SPR) biosensors. While chemiluminescence describes the generation of light due to release of energy during a chemical reaction, SPR measures changes in refractive index caused by structural alterations in the vicinity of a thin film metal surface [36]. The numerous chemiluminescence (CL) applications in microfluidic analysis systems using immobilized enzymes, antibodies or nucleic acids have been recently described [37-39]. In turn, electroanalytical methods are highly compatible with micro- and nanomachining (MEMS) technology and can be segmented into current (amperometric), potential (potentiometric) or impedance (impediometric) techniques [40-43]. Evolving from ISFETs, a recent technology combines potentiometry and optical detection, known as light addressable potentiometric sensor (LAPS), that can be used for the detection of pathogen *E. coli* [44]. Alternative detection methods for pathogen sensing include the application of silver dots for direct optical density measurements using a scanometric reader [45,46], or biosensors using resonance light scattering (RLS) techniques based on nanometer-sized metallic particles (mostly gold) covalently linked to antibodies. These metal colloidal particles radiate energy in the form of scattered light when illuminated by a white light source [47]. Altogether, LOC devices present themselves as a flexible technology platform that can be readily adapted to specific identification needs. A whole range of materials and mode of detection can be specifically selected for either low cost applications or high end analysis. Having reviewed the various materials and detection methods employed in lab-on-a-chip devices, we now provide a detail list of LOC studies grouped by class of target analytes.

## 3. Nucleic Acid Based Microfluidic Pathogen Sensing

The analysis of conserved DNA or RNA sequences using PCR and RT-PCR techniques has been extensively used to detect infectious diseases and to determine the stage of actual disease [8]. Although this review focuses on the application of microfluidic biochips (LOC) for pathogen sensing, it is important to note that microfluidics has also been applied to microarray technology [48,49]. In contrast to LOC devices consisting of a network of microchannel and reaction chambers, microarrays are generally described as a multiplex technology consisting of an arrayed series of thousands of microscopic spots of DNA oligonucleotides covalently attached on a solid support to determine the relative abundance of nucleic acid sequences in the target. Examples of integrated microfluidic-microarray technology include the identification of *Bacillus* species, influenza, *Yersinia enterocolitica* and fungal pathogens [50-53]. As a technology, nucleic acid detection has been proven to be very sensitive and specific due to target amplification and base-pairing interactions. Additionally, high-throughput systems for rapid and parallelized detection of nucleic acids identifying specific bacterial pathogens have been reported [54]. In regards to LOC devices, DNA based pathogen detection can be achieved by direct target probing or after target amplification. Since minimum detection levels vary between 10^5^-10^6^ target molecules, direct target probing using hybridization-based assays are limited in terms of sensitivity, thus requiring additional signal enhancement techniques. One of the enhancement techniques include the bead-based methods [55,56] that reduce diffusion time and increase biorecognition events [57]. The application of magnetic forces can also be used to discriminate between specific and non-specific binding leading to increased selectivity and increased selectivity [58]. Another widely applied method to accurately detect small amounts of infectious pathogens includes target amplification techniques. Here amplifications leading to increased sensitivity can be obtained through polymerase chain reaction (PCR), ligase chain reaction (LCR) or nucleic acid sequence based amplification (NASBA) [59]. Overall, micro-PCR chips can be classified into three categories including (i) stationary-chamber micro PCR-chips as nano/picoliter reservoir for conventional thermocycling, (ii) continuous-flow micro-PCR chips where different temperature zones are established at different locations and the sample is moved between individual temperature zones for cycling [60], and (iii) droplet-based PCR systems where amplification reactions are conducted in water-in-oil droplets for each amplicon [61]. A general problem found with LOC devices is unspecific adsorption due to the large surface-to-volume ratios present in microchannels [62], that is known to inhibit PCR reactions. However, a variety of specific surface modification procedures [63,64] or bulk modification methods for polymers have been recently implemented to overcome this limitation [65,66]. Below we will outline how, despite the overall complexity of DNA analysis involving sample preparation, DNA isolation, amplification and detection, multiple procedures and functional components have been successfully integrated into a single biochip.

### 3.1. Sample preparation, isolation, amplification and detection of pathogenic DNA/RNA

Although a sample (pre)-preparation step is not always necessary for successful PCR amplification [56], it is often required when using environmental or otherwise complex samples [64]. This is particularly true for the identification of pathogens in food samples [67], whole blood [68], urine [69,70], wastewater [71] and others [48,62,72-74]. Consequently, with LOC devices, sample pre-treatment has routinely been combined with DNA/RNA isolation procedures. Popular isolation approaches include pathogen capture using antibody labelled magnetic beads [75] or elektrokinetic capture of bacterial cells such as the dielectrophoretic capture of malarial-parasitized cells [72,76]. Cell lyses and PCR analysis can be accomplished chemically or optically. Examples of optical approaches include the Laser-Irradiated Magnetic Bead system (LIMBS), which combines optical forces with magnetic beads for direct cell lyses and DNA capture [73]. Other optical methods utilize optothermal properties of nanoparticles to transform near infrared light energy into thermal energy for pathogen lyses [62]. Following nucleic acid isolation, direct target detection or micro-sized PCR, also called PCR microfluidic chip, is seen as the next step in the development of integrated micro-total analysis system (μTAS) [55,77]. Various reviews on the integration of PCR reaction in microfluidic platforms, not specialized to pathogen sensing, have been published elsewhere [64,78]. However, reverse-transcriptase PCR [66], real-time reverse transcription PCR [79], limited dilution PCR [80] and real-time PCR [81] have been specifically applied to nucleic acid based microfluidic pathogen sensing. Other amplification based methods include the application of immobilised primers for bacterial DNA detection [74], the combination of on-chip PCR followed by microarray-based fluorescence detection [82], and the application of field-effect transistors for label free detection of bacterial DNA [83]. In many instances capillary electrophoresis is employed to separate the amplicon and primers prior to detection [70,84]. While a variety of DNA capture, isolation and amplification procedures have been successfully integrated into LOC devices, applied detection techniques are limited to optical methods. Although fluorescence detection dominates the field of DNA detection, a variety of electrochemical and magnetoresistive sensors have also been successfully integrated in microfluidic based nucleic acid detection devices [67,69,85,86].

## 4. Microfluidic Protein/Enzyme Based Pathogen Sensing

Another powerful analytical tool for pathogen detection employs immunological methods that rely on the specific affinities of protein-protein, protein-carbohydrate or protein-DNA interactions [2]. Antigen (Ag)/antibody (Ab) recognition systems are, for instance, well understood and widely accepted for pathogen detection. One example of a highly integrated portable antibody based pathogen chip was recently presented involving a magnetoresistive immunosensor in a four channel configuration for the detection of enterohemorrhagic *E. coli* (EHEC) [21]. Although antibodies, polyclonal Abs (pAb) or monoclonal Abs (mAb) can be readily obtained, a major drawback of antibodies includes quality-assured preparation, which is an important aspect for any analytical method. Alternatively, recombinant antibody-fragments (rAbs), such as single chain variable fragments (scFv) and Fabs, have gained increasing popularity due to comparable specificity but much lower cost of production [87]. As an example, single domain antibodies obtained from cartilaginous fish have shown great promise for POC applications due to their good solubility and excellent thermal stability [88]. A detailed review on antibody fragments as probes in biosensors can be found elsewhere [89]. Independent from the type of affinity capture utilized, the biorecognition layer is generally immobilized on a solid support. Since most microfluidic pathogen sensing systems are based on polymeric materials, such as poly-(methylmethacrylate) (PMMA) and polycarbonate (PC), poly(dimethylsiloxane) (PDMS) surface modifications are required to introduce functional groups for protein coupling [90]. Sensor surface functionalization is either achieved through covalent attachment using affinity tags such as poly-amino acids, protein G/A, biotin and recombinant fusion proteins or simply by physisorption [91,92]. Additionally, supported bilayer membranes (SBMs) have been applied to minimize non-specific adsorption of biomolecules [93]. Also, self-assembled protein-microarrays have been generated through contact-printing of complementary DNA onto glass slides followed by translating the target proteins with mammalian reticulocyte lysate [94,95]. Overall, self-assembling technologies are currently adapted to microfluidic devices [96,97] and recent advances in the rapid generation of protein arrays are reviewed in He *et al.* [97]. A drawback of protein based pathogen recognition systems is the need to preserve the native protein state for optimal orientation of the protein-target interaction [98,99] after immobilization. Poor binding-site recognition results in decreased sensitivity and reusability [100]. However, a number of amplification techniques, such as the conjugation of additional enzymes [101] or liposomes encapsulating fluorescence dyes or electroactive compounds, have been successfully demonstrated to increase sensor sensitivity [102-104]. Another drawback for most LOC devices involves the recycling of Ab/Ag-based recognition systems [105]. Here molecular imprinted polymers may offer a real alternative to antibodies due to their inherent robustness and reproducibility [106]. Another alternative to classic affinity capture methods are enzyme-substrate reactions that have the advantage of auto-regeneration of the binding site without any affinity or specificity loss over a large number of cycles. For instance, several toxin sensors have been realized based on the enzymatic cleavage of a known immobilized substrate or based on enzyme inhibition by a toxin [107,108]. Additionally, a microfluidic-chip for the detection of pathological prion proteins based on enzyme-grafted magnetic beads has been developed [109]. Since antibody based pathogen sensors are predominantly used in combination with microfluidics, some of the more recent developments will be discussed in further detail in the subsection below.

### 4.1. Microfluidic pathogen detection systems based antibody- and aptamer sensor

As mentioned in the above section, the application of antibody based recognition systems is still dominant in the field of microfluidic pathogen sensing. Some of the recent highlights include the combination of electrochemical and optical or label free detection techniques, nanotechnology advanced detection systems and antibody microarray systems. An example is the detection of Cholera toxin subunit B (CTB) using electrochemical and fluorescence based microfluidic biosensors [117]. Here a combination of CTB-antibodies and Ganglioside GM1, the natural target of the CTB, were used as a specific recognition system. Another recent development is a direct-charge transfer (DCT) immunosensor based on antibody recognition, in combination with conducting polymers (e.g. polyaniline) as transducers, for the detection of different *Bacillus* species [119]. The working principle involves an antibody-antigen-antibody sandwich design and DCT to generate a resistance signal capable of detecting concentrations as low as 100 CFU/mL. The speed, sensitivity and ease-of-use make this disposable biosensor a promising device for rapid POC-detection of foodborne pathogens. One more advancement is the multiplex detection of different pathogens using quantum dot barcodes conjugated to targeting antibodies within an electrokinetically driven microfluidics and photon counting detection system [114]. Another “nano-on-micro” approach for LOC-immunoassay implements quantum-dots (QDs) conjugated to microspheres to enable multiplexed detection of analytes (e.g. up to 10 different inflammatory proteins) using microsphere light-scattering for detection [115,116]. Alternatively, the application of magnetic beads and fluidic force discrimination (FFD) for antibody based pathogen detection has shown multiplexed detection capability using two proteins ricin A chain (RCA) and staphylococcal enterotoxin B (SEB) [55]. In FFD assays, analytes are captured and labelled by microbeads while a controlled laminar flow is used to apply fluid mechanical forces sufficient to remove only non-specifically bound beads. The density of beads that remain bound to the microarray surface is proportional to the analyte concentration [118]. Delehanty *et al.* developed an antibody microarray system with continuous fluid flow-through capable to detect microbial toxins [118]. They achieved simultaneous detection of cholera toxin and staphylococcal enterotoxin B within 15 min at levels as low as 8 and 4 ng/mL using fluorescent-labled antibodies and scanning confocal microscopy.

In contrast to antibodies, aptamers are generated by an *in-vitro* selection process referred to as systematic evolution of ligands by exponential enrichment (SELEX) [110,111]. These specific nucleic acid sequences represent a promising alternative to antibodies as recognition agents since the generation of poly- or monoclonal antibodies is often challenging and time-consuming. The application of aptamer sensors for the detection of microbial and viral pathogens has been recently reviewed [112]. One example of aptamers successfully applied as biorecognition elements involves the application of single-walled carbon nanotubes (SWNT) as transducers [113]. Altogether, a variety of different detection methods have been implemented for signal generation including electrical conductance methods [121,122], optical detection [123], ATP-bioluminescence [124], and mass-sensitive systems [125].

## 5. Microfluidic Cell Based Pathogen Sensing

In contrast to sensing DNA or proteins that indicate the presence of pathogens, cell based assays allow for direct identification, differentiation and quantitation of clinical relevant cellular systems. One of the earliest applications of microfluidics to cell analysis involves flow cytometers for cell counting, several of which are commercially available today (e.g. Agilent 2100 bioanalyzer) [126,127]. The application of cytometers to count erythrocytes and CD4^+^ cells is particularly important because it allows the monitoring of the progression of HIV infections for AIDS patients [8,128]. More recently, a microfluidic based 10-channel capillary chip coated with selected capture antibodies was also used to detect a variety of pathogens based on chemiluminescence immunoassay (EIA) [129]. The controlled fluid flow through capillaries and microchannels can be generally achieved through hydrodynamic (e.g. pressure driven) or electrokinetic flow switching and dielectrophoresis [31,130]. The application of electric fields in microfluidics is also significant because it led to continuous cell separation systems capable to trapping bacteria or discriminating between dead and live yeast [131-133]. Another promising cell-based application of LOC devices for medical diagnostics is the miniaturization of microbiological culture assays to identify drug-resistant bacterial strains [134,135]. Despite of all these recent advances, there is still a need for miniature, low-cost and portable sensors capable of the rapid detection and accurate identification of bacteria in complex matrices. Traditional detection methods require the growth of a single bacteria into colonies in different types of media, followed by a lengthy identification process involving morphological and biochemical testing [136,137]. Additionally, serological characterization based on the determination of antigens expressed on the bacterial cell surface is of importance in microbiological diagnostics [138]. Although conventional immunoassays are labour-intensive, they have become the main analytical technique used to study infectious diseases due to the high sensitivity and selectivity of the antigen-antibody reaction [139]. Consequently the integration of immunoassays in microfluidic devices is most commonly applied for cell based pathogen detection. In particular, the high surface to volume ratio found in microchannels is ideally suited to selectively functionalize surfaces with capture agents [140]. Here microfluidic devices take advantage of the significantly increased probability of pathogen interaction and cell capture at modified/activated sensor surfaces along the flow pathway that allows for the identification of small amounts of pathogens in a short period of time. Antibody/antigen recognition systems employed for cell-based microfluidic pathogen sensing has been demonstrated using fluorescence [32], chemiluminescence [129], optical leaky waveguides [20], surface plasmon resonance [141], impedance [29,142], love acoustic waves [24], and conducting polymers [143]. Another alternative cell capture and identification method of microorganism involves the covalent attachment of peptide ligands following intrinsic fluorescence detection [25]. Although immunoassays offer a high degree of selectivity in many instances, additional signal amplification is required to detect small amounts of pathogens. This can either be achieved through enzymatic signal amplification [21,23], or through pre-concentrations steps including dielectrophoresis [26,133], ultrasonic deposition of cells [144], magnetic beads [59,145,146] and membrane filters [147]. The identification of pathogenic microorganisms using LOC devices has shown great promise, mostly because the sample preparation procedure is dramatically reduced and the pathogen can be directly detected translating into increased speed and accuracy. However, in addition to the previously discussed disadvantages of antibody-based detection systems, the stringent detection limits requiring the identification of a single cell in 100 mL sample still inhibit more frequent commercial applications.

## 6. Commercial LOC Based Pathogen Sensor Systems

Due to the pressing need to rapidly detect pathogens, a variety of commercial tools have been developed to overcome existing diagnostic challenges. Below we have listed a variety of companies that offer chip based pathogen sensing systems using different off-chip and on-chip detection methods.

For instance, the CANARY (cellular analysis and notification of antigen risks and yield) biosensor is a B-Lymphocytes based antigen-detection device which demonstrated rapid screening of a variety of pathogens at low level [30]. Additionally, SPR based biosensors are currently implemented to be applied in field-deployable devices sensing of small molecules, proteins, viruses and whole microbes using a 24-channel SPREETA (Sensata) sensor unit [36]. Invitrogen has developed a device for Multi-Agent Portable Pathogen Detection System (MAPP-DS) based on a unique, multiplexed immunoassay and RLS detection.

Automated protocols for the POC detection of *Bacillus anthracis*, *Bacillus subtilis*, staphylococcal enterotoxin B, Clostridum botulinum toxin A, *Yersinia pestis*, and ricin A chain are available. According to the manufacturer's specifications, limits of detection are generally 1-2 log units better than ELISA and up to 3 log units better than lateral flow assays.

Table 1 clearly shows that most commercialized pathogen detection technologies are DNA based. This development has been facilitated by the successful application of microfluidics in the genomics research area. However, it can be expected that broader commercialization of protein chips, including microfluidic application, will benefit protein-based microfluidic pathogen detection systems in the near future.

## 7. Conclusions

In this review we discuss the latest advances, commercial applications and future trends of pathogen sensing methods combined with microfluidic systems. Research on microfluidic based pathogen sensing systems is still a young and growing field within LOC devices. Consequently, microchip technology presents itself as a flexible detection platform that can be readily adapted to specific pathogen related needs. These include low detection limits, complex sample matrices and device portability. The range of materials and detection modes to choose from allow, in principle, for the development of low cost, fast and rapid LOC devices for point of care diagnostics. However, many systems have so far only been tested using simple samples consisting of pure cultures in a laboratory setting. Since LOC based pathogen sensors compete with laboratory-scale technologies in the analysis of complex biological samples, only highly integrated microdevices (μTAS) will find real world applications. The analysis of biological samples translates into several processing steps such as sample preparation, analyte enrichment, labelling, signal amplification and detection to be performed on chip [1]. So far only a few micro-total analysis systems (μTAS) capable of delivering results from complex biological samples in a single system have been developed [68,70,73-75]. The main application of microfluidics in pathogen detection involves DNA based methods. In this case, a very promising approach for future applications involves the combination of real-time PCR and microarray technologies (Real-Time Array PCR) that allow multiplex pathogen detection. We have also discussed how antibody based affinity capture systems dominate protein-based microfluidic pathogen sensing methods. However, new advances in capture agent research will greatly benefit future developments. For instance, aptamer-based sensors present themselves as attractive alternatives to antibodies due to their relative ease of isolation and modification as well as intrinsic resistance against denaturation. The application of apatmers as selective capture agents can be used for the detection of microbial and viral pathogens. Cell based assays also have advantages because they discriminate between live and dead pathogens and allow for the rapid identification of small amounts of bacteria. In summary, the large number of publications found in high impact journals and the availability of several commercial devices, indicate that microfluidic applications in the life sciences have become mainstream. The trend points towards near-patient testing using faster, portable and more accurate diagnostic methods and devices. It is therefore concluded by the authors that next generation of pathogen sensing developments will be facilitated by advances in LOC devices.

## Figures and Tables

**Table 1.** Commercial available chip based pathogen sensing systems.

**Company**	**Target**	**Website**
Advanced Liquide Logics	Immunoassay	www.liquid-logic.com
Cepheid	DNA	www.cepheid.com
CombiMatrix CustomArray	DNA, biological threads	www.combimatrix.com
Invitrogen	DNA	www.invitrogen.com
Affymetrix	DNA	www.affymetrix.com
Caliper	DNA	www.caliperls.com
Febit	DNA/RNA/Proteins	www.febit.com
Claros Diagnostics	Proteins	www.clarosdx.com
HandyLab	DNA, proteins	www.handylab.com
Abbott Diagnostics (iStat)	Markers	www.istat.com
LabNow	HIV/AIDS	www.labnow.com
Micronics	Enteric pathogens	www.micronics.net
Nanogen	DNA/RNA	www.nanogen.com
Nanosphere	DNA, proteins	www.nanosphere-inc.com
Sensata (Spreeta)	Viruses, bacteria	www.sensata.com
Sequella	Proteins	www.sequella.com
BIAcore	Bacteria, viruses	www.biacore.com
Canary	B-lymphocytes	www.canarysystem.com
Rapid Plex (ICx Biosystems)	Bacteria, protein	www.inivtrogen.com

## References

[1] Yager P., Edwards T., Fu E., Helton K., Nelson K., Tam M.R., Weigl B.H. (2006). Microfluidic Diagnostic Technologies for Global Public Health. Nat. Biotechnol..

[2] Lazcka O., Del Campo F.J., Muñoz F.X. (2007). Pathogen Detection: A Perspective of Traditional Methods and Biosensors. Biosens. Bioelectron..

[3] Gomez R., Bashir R., Sarikaya A. (2001). Microfluidic Biochip for Impedance Spectroscopy of Biological Species. Biomed. Microdevices.

[4] Auroux P.A., Iossifidis D., Reyes D.R., Manz A. (2002). Micro Total Analysis Systems. 2. Analytical Standard Operations and Applications. Anal. Chem..

[5] Reyes D.R., Iossifidis D., Auroux P.A., Manz A. (2002). Micro Total Analysis Systems. 1. Introduction, Theory, and Technology. Anal. Chem..

[6] Dittrich P.S., Tachikawa K., Manz A. (2006). Micro Total Analysis Systems. Latest Advancements and Trends. Anal. Chem..

[7] Vilkner T., Janasek D., Manz A. (2004). Micro Total Analysis Systems. Recent Developments. Anal. Chem..

[8] Chin C.D., Linder V., Sia S.K. (2007). Lab-on-a-Chip Devices for Global Health: Past Studies and Future Opportunities. Lab Chip.

[9] Morens D.M., Folkers G.K., Fauci A.S. (2004). The Challenge of Emerging and Re-emerging Infectious Diseases. Nature.

[10] Harrison D.J., Manz A., Fan Z.H., Ludi H., Widmer H.M. (1992). Capillary Electrophoresis and Sample Injection Systems Integrated on a Planar Glass Chip. Anal. Chem..

[11] Beebe D.J., Moore J.S., Yu Q., Liu R.H., Kraft M.L., Jo B.H., Devadoss C. (2000). Microfluidic Tectonics: A Comprehensive Construction Platform for Microfluidic Systems. Proc. Nat. Acad. Sci. U.S.A..

[12] Lagally E.T., Simpson P.C., Mathies R.A. (2000). Monolithic Integrated Microfluidic DNA Amplification and Capillary Electrophoresis Analysis System. Sens. Actuat. B.

[13] Ertl P., Emrich C.A., Singhal P., Mathies R.A. (2004). Capillary Electrophoresis Chips with a Sheath-flow Supported Electrochemical Detection System. Anal. Chem..

[14] Whitesides G.M. (2003). The ‘Right’ Size in Nanobiotechnology. Nat. Biotechnol..

[15] Ionescu-Zanetti C., Shaw R.M., Seo J., Jan Y.N., Jan L.Y., Lee L.P. (2005). Mammalian Electrophysiology on a Microfluidic Platform. Proc. Nat. Acad. Sci. U.S.A..

[16] Becker H., Gärtner C. (2008). Polymer Microfabrication Technologies for Microfluidic Systems. Anal. Bioanal. Chem..

[17] Mukhopadhyay R. (2007). When PDMS Isn't the Best. Anal. Chem..

[18] Duffy D.C., McDonald J.C., Schueller O.J.A., Whitesides G.M. (1998). Rapid Prototyping of Microfluidc Systems in Poly(dimethylsiloxane). Anal. Chem..

[19] Abgrall P., Gue A.M. (2007). Lab-on-a-Chip Technologies: Making a Microfluidic Network and Coupling It into a Complete Microsystem — A Review. J. Micromech. Microeng..

[20] Zourob M., Mohr S., Brown B.J.T., Fielden P.R., McDonnell M.B., Goddard N.J. (2005). Bacteria Detection Using Disposable Optical Leaky Waveguide Sensors. Biosens. Bioelectron..

[21] Mujika M., Arana S., Castano E., Tijero M., Vilares R., Ruano-Lopez J.M. (2009). Magnetoresisitve Immunosensor for the Detection of *Escherichia coli* O157:H7. Biosens. Bioelectron..

[22] Li Y., Su X. (2006). -L. Microfluidics-based Optical Biosensing Method for Rapid Detection of *Escherichia coli* O157:H7. J. Rapid Methods Autom. Microbiol..

[23] Godber B., Kevin S.J., Thompson K.S.J., Rehak M., Uludag Y., Kelling S., Sleptsov M., Frogley M., Wiehler K., Whalen C., Cooper J.M. (2005). Direct Quantification of Analyte Concentration by Resonant Acoustic Profiling. Clin. Chem..

[24] Tamarin O., Comeau S., Dejous C., Moynet D., Riebre D., Beziam J. (2003). Real Time Device for Biosensing: Design of a Bacteriophage Model Using Love Acoustic Waves. Biosens. Bioelectron..

[25] Mason H.-Y., Lloyd C., Dice M., Sinclair R., Ellis W., Powers L. (2003). Taxonomic Identification of Microorganisms by Capture and Intrinsic Fluorescence Detection. Biosens. Bioelectron..

[26] Gomez-Sjoberg R., Morisette D.T., Bashir R. (2005). Impedance Microbiology-on-a-Chip: Microfluidic Bioprocessor for Rapid Detection of Bacterial Metabolism. J. Microelectromech. Syst..

[27] Lee S.J., Park J.S., Im H.T., Jung H.-I. (2008). A Microfluidic ATP-bioluminescence Sensor for the Detection of Airborne Microbes. Sens. Actuat. B.

[28] Silley P., Forsythe S. (1996). Impedance Microbiology — A Rapid Change for Microbiologists. J. Appl. Bacteriol..

[29] Boehm D.A., Gottlieb P.A., Hua S.Z. (2007). On-chip Microfluidic Biosensors for Bacterial Detection and Identification. Sens. Actuat. B.

[30] Rider T.H., Petrovick M.S., Nargi R.E., Harper J.D., Schwoebel E.D., Mathews R.H., Blanchard D.J., Bortolin L.T., Young A.M., Chen J., Hollis M.A. (2003). A B Cell-based Sensor for Rapid Identification of Pathogens. Science.

[31] Xiang Q., Hu G., Gao Y., Li D. (2006). Miniaturized Immunoassay Microfluidic System with Electrokinetic Control. Biosens. Bioelectron..

[32] Yadavalli V.K., Pishko M.V. (2004). Biosensing in Microfluidic Channels Using Fluorescence Polarization. Anal. Chim. Acta.

[33] Ivnitski D., Abdel-Hamid I., Atanasov P., Wilkins E., Stricher S. (2000). Application of Electrochemical Biosensors for Detection of Food Pathogenic Bacteria. Electroanalysis.

[34] Yi C., Zhang Q., Li C.W., Yang J., Zhao J., Yang M. (2006). Optical and Electrochemical Detection Techniques for Cell-based Microfluidic Systems. Anal. Bioanal.Chem..

[35] Gaits F., Hahn K. (2003). Shedding Light on Cell Signaling: Interpretation of FRET Biosensors. Sci STKE.

[36] Chinowsky T.M., Quinn J.G., Bartholomew D.U., Kaiser R., Elkind J.L. (2003). Performance of the Spreeta 2000 Integrated Surface Plasmon Resonance Affinity Sensor. Sens. Actuat. B.

[37] Nakamura H., Murakami Y., Yokoyama K., Tamiya E., Karube I. (2001). A Compactly Integrated Flow Cell with a Chemiluminescent FIA System for Determining Lactate Concentration in Serum. Anal. Chem..

[38] Yakovleva J., Davidsson R., Lobanova R., Bengtsson M., Eremin S., Laurell T., Emneus J. (2002). Microfluidic Enzyme Immunoassay Using Silicon Microchip with Immobilized Antibodies and Chemiluminescence Detection. Anal. Chem..

[39] Wang Y., Vaidya B., Farquar H.D., Stryjewski W., Hammer R.P., McCarley R.L., Soper S.A., Cheng Y.W., Barany R. (2003). Microarrays Assembled in Microfluidic Chips Fabricated from Poly(methyl methacrylate) for the Detection of Low-Abundant DNA Mutations. Anal. Chem..

[40] Spegel C., Heiskanen A., Skjolding L.H.D., Emneus J. (2008). Chip Based Electroanalytical Systems for Cell Analysis. Electroanalysis.

[41] Ertl P., Wagner M., Corton E., Mikkelsen S.R. (2003). Rapid Identification of Viable *Escherichia coli* subspecies with an Electrochemical Screen-printed Biosensor Array. Biosens. Bioelectron..

[42] Sadik O.A., Aluoch A.A., Zhou A. (2009). Status of Biomolecular Recognition Using Electrochemical Techniques. Biosens. Bioelectron..

[43] Sandifer J.R., V J. (1999). Review of Biosensor and Industrial Applications of pH-ISFETs and an Evaluation of Honeywell's “DuraFET”. Microchim. Acta.

[44] Gehring A.G., Patterson D.L., Tu S.I. (1998). Use of a Light-addressable Potentiometric Sensor for the Detection of *Escherichia coli* O157:H7. Anal. Biochem..

[45] Taton T.A., Lu G., Mirkin C.A. (2001). Two-color Labeling of Oligonucleotide Arrays via Size-Selective Scattering of Nanoparticle Probes. J. Am. Chem. Soc..

[46] Taton T.A., Mirkin C.A., Letsinger R.L. (2000). Scanometric DNA Array Detection with Nanoparticle Probes. Science.

[47] Shang L., Chen H., Deng L., Dong S. (2008). Enhanced Resonance Light Scattering Based on Biocatalytic Growth of Gold Nanoparticles for Biosensors Design. Biosens. Bioelectron..

[48] Anderson R.C., Su X., Bodgan G.J., Fenton J. (2000). A Miniature Integrated Device for Automated Multistep Genetic Assays. Nucleic Acids Res..

[49] Situma C., Hashimoto M., Soper S.A. (2006). Mergin Microfluidics with Microarray-based Bioassays. Biomol. Eng..

[50] Liu R.H., Munro S.B., Nguyen T., Siuda T., Suciu D., Bizak M., Slota M., Fuji H.S., Danley D., McShea A. (2006). Integrated Microfluidic Custom Array Device for Bacterial Genotyping and Identification. J. Assoc. Lab. Autom..

[51] Wang L., Li P.C.H., Yu H.-Z., Parameswaran A.M. (2008). Fungal Pathogenic Nucleic Acid Detection Achieved with a Microfluidic Microarray Device. Anal. Chim. Acta.

[52] Liu R.H., Lodes M.J., Nyuyen T., Siuda T., Slota M., Fuji H.S., McShea A. (2006). Validation of a Fully Integrated Microfluidic Array Device for Influenza A subtype Identification and Sequencing. Anal. Chem..

[53] Myers K.M., Gaba J., Al-Khaldi S.F. (2006). Molecular Identification of *Yersinia enterocolitica* Isolated from Pasteurized Whole Milk Using DNA Microarray Chip Hybridization. Mol. Cell. Probes.

[54] Piliarik M., Paravoa L., Homola J. (2009). High-throughput SPR Sensor for Food Safety. Biosens. Bioelectron..

[55] Mulvaney S.P., Cole C.L., Kniller M.D., Malito M., Tamanaha C.R., Rife J.C., Stanton M.W. (2007). Rapid, Femtomolar Bioassays in Complex Matrices Combining Microfluidics and Magnetoelectronics. Biosens. Bioelectron..

[56] Gabig-Ciminiska M., Andresen H., Albers J., Hintsche R., Enfors S.O. (2004). Identification of Pathogenic Microbial Cells and Spores by Electrochemcial Detection on a Biochip. Microbial Cell Factories.

[57] Chang H.-C. (2007). Nanobead Electrokinetics: the Enabling Microfluidic Platform for Rapid Multi-Target Pathogen Sensing. AICHE J..

[58] Edelstein R.L., Tamanaha C.R., Sheehan P.D., Miller M.M., Baselt D.R., Whitman L.J., Colton R.J. (2000). The BARC Biosensor Applied to the Detection of Biological Warfare Agents. Biosens. Bioelectron..

[59] Zaytseva N.V., Goral V.N., Montagna R.A., Baeumner A.J. (2005). Development of a Microfluidic Biosensor Module for Pathogen Detection. Lab Chip.

[60] Wang C.-H., Chen Y.Y., Liao C.S., Hsieh T.M., Luo C.H., Wu J.J., Lee H.H., Lee G.B. (2007). Circulating Polymerase Chain Reaction Chips utilizing Multiple-membrane Activation. J. Micromech. Microeng..

[61] Chang Y.-H., Lee G.B., Huang F.C., Chen Y.Y., Lin L. (2006). Integrated Polymerase Chain Reaction Chips Utilizing Digital Microfluidics. Biomed. Microdevices.

[62] Cheong K.H., Yi D.K., Lee J.-G., Park J.M., Kim M.J., Edel J.B., Ko C. (2008). Gold Nanoparticles for One Step DNA Extraction and Real-time PCR of Pathogens in a Single Chamber. Lab Chip.

[63] Christensen T.B., Bang D.D., Wolff A. (2008). Multplex Polymerase Chain Rreaction (PCR) on a SU-8 Chip. Microelectron. Eng..

[64] Zhang C., Xu J., Ma W., Zheng W. (2006). PCR Microfluidic Devices for DNA Amplification. Biotechnol. Adv..

[65] Muck A., Svatos A. (2007). Chemical Modification of Polymeric Microchip Devices. Talanta.

[66] Felbel J., Reichert A., Kielpinski M., Urban M., Henkel T., Häfner N., Dürst M., Weber J. (2008). Reverse Transcription-Polyermerase Chain Reaction (RT-PCR) in Flow-through Micro-reactors: Thermal and Fluidic concepts. Chem. Eng. J..

[67] Liu Y.L., Elsholz B., Enfors S.-O., Gabig-Ciminiska M. (2007). Confirmative Electric DNA Array-based Test for Food Poisoning Bacillus cereus. J. Microbiol. Methods.

[68] Easley C., Karlinseq J.M., Bienvenue J.M., Legendre L.A., Roper M.G., Feldman S.H., Hughes M.A., Hewlett E.L., Merkel T.J., Ferrance J.P., Landers J.P. (2006). A Fully Integrated Microfuidic Genetic Analysis System with Sample-in-Answer-out Capability. Proc. Nat. Acad. Sci. U.S.A..

[69] Liao J.C., Mastali M., Gau V., Suchard M.A., Moller A.K., Bruckner D.A., Babbitt J.T., Gornbein J., Landaw E.M., McCabe E.R.B., Churchill B.M., Haake D.A. (2006). Use of Electrochemical DNA Biosensors for Rapid Molecular Identification of Uropathogens in Clinical Urine Specimens. J. Clin. Microbiol..

[70] Kaigala G.V., Huskins R.J., Preiksaitis J., Pang X.-L., Pilarski L.M., Backhouse C.J. (2006). Automated Screening Using Microfluidic Chip-based PCR and Product Detection to Assess Risk of BK Virus-associated Nephropathy in Renal Transplant Recipients. Electrophoresis..

[71] Gilbride K.A., Lee D.-Y., Beaudette L.A. (2006). Molecular Techniques in Wastewater: Understanding Microbial Communities, Detecting Pathogens and Real-time Process Control. J. Microbiol. Methods.

[72] Bhattacharya S., Salmat S., Morisette D., Banada P., Akin D., Liu Y.-S., Bhunia A.K., Ladisch M., Bashir R. (2008). PCR-based Detection in a Micro-fabricated Platform. Lab Chip.

[73] Lee J.-G., Cheong K.H., Huh N., Kim S., Choi J.W., Ko C. (2006). Microchip-Based One Step DNA Extraction and Real-time PCR in one Chamber for Rapid Pathogen Identification. Lab Chip.

[74] Anzai Y., Saito S., Fujimoto K., Kinoshita K., Kato F. (2008). Detection and Identification of Species with Bacterial Cells Using a Plastic DNA Array. J. Health Sci..

[75] Lien K.-Y., Lee W.C., Lei H.Y., Lee G.B. (2007). Integrated Reverse Transcription Polymerase Chain Reaction Systems for Virus Detection. Biosens. Bioelectron..

[76] Gascoyne P., Satayavivad J., Ruchirawat M. (2004). Microfluidic Approaches to Malaria Detection. Acta Tropica.

[77] Liao C.-S., Lee G.B., Liu H.S., Hsieh T.M., Luo C.H. (2005). Minaturized RT-PCR System for Diagnosis of RNA-based Viruses. Nucleic Acids Res..

[78] Roper M.G., Easley C.J., Landers J.P. (2005). Advances on Polymerase Chain Reaction on Microfluidic Chips. Anal. Chem..

[79] Beer N.R., Wheeler E.K., Lee-Houghton L., Watkins N., Nasarabidi S., Hebert N., Leung P., Arnold D.W., Bailey C.G., Colston B.W. (2008). On-chip Single-copy Real-time Reverse-Transcription PCR in Isolated Picoliter Droplets. Anal. Chem..

[80] Kiss M.M., Ortoleva-Donnelly L., Beer N.R., Warner J., Bailey C.G., Colston B.W., Rothberg J.M., Link D.R., Leamon J.H. (2008). High-throughput Quantitative Polymerase Chain Reaction in Picoliter Droplets. Anal. Chem..

[81] Csordas A., Delwiche M.J., Barak J.D. (2008). Nucleic Acid Sensor and Fluid Handling for Detection of Bacterial Pathogens. Sens. Actuat. B.

[82] Mitterer G., Huber M., Leidinger E., Kirisits C., Lubitz W., Mueller M.W., Schmidt W.M. (2004). Microarray-based Identification of Bacteria in Clinical Samples by Solid-Phase PCR Amplification of 23S ribosomal DNA Sequences. J. Clin. Microbiol..

[83] Hou C.-S.J., Godin M., Payer K., Chakarabarti R., Manalis S.R. (2007). Integrated Microelectronic Device for Label-free Nucleic Acid Amplification and Detection. Lab Chip.

[84] Huang F.-C., Liao C.S., Lee G.B. (2006). An Integrated Microfluidic Chip for DNA/RNA Amplification, Electrophoresis, Separation and On-line Optical Detection. Electrophoresis..

[85] Möller R., Schüler T., Günther S., Carlsohn R., Munder T., Fritzsche W. (2008). Electrical DNA-Chip-based Identification of Different Species of the Genus Kitasatospora. Appl. Microbiol. Biotechnol..

[86] Yeung S.-W., Lee T.M.-H., Cai H., Hsing I.M. (2006). A DNA Biochip for On-the-spot Multiplexed Pathogen Identification. Nucleic Acids Res..

[87] Holliger P., Hudson P.J. (2005). Engineered Antibody Fragments and the Rise of Single Domains. Nat. Biotechnol..

[88] Liu J.L., Anderson G.P., Goldman E.R. (2007). Isolation of Anti-toxin Single Domain Antibodies from a Semi-synthetic Spiny Dogfish Shark Display Library. BMC Biotechnol..

[89] Saerens D., Huang L., Bonroy K., Muyldermans S. (2008). Antibody Fragments as Probe in Biosensor Development. Sensors..

[90] Makamba H., Kim J.H., Lim K., Park N., Hahn J.H. (2003). Surface Modification of Poly(dimethylsiloxane) Microchannels. Electrophoresis..

[91] Talapatra A., Rouse R., Hardiman G. (2002). Protein Microarrays: Challenges and Promises. Pharmacogenomics..

[92] Seurynck-Servoss S.L., Baird C.L., Rodland K.D., Zangar R.C. (2007). Surface Chemistries for Antibody Microarrays. Front. Biosci..

[93] Dong Y., Phillips K.S., Cheng Q. (2006). Immunosensing of Staphylococcus Enterotoxin B (SEB) in Milk with PDMS Microfluidic Systems Using Reinforced Supported Bi-layer Membranes (r-SBMs). Lab Chip.

[94] Ramachandran N., Hainsworth E., Bhullar B., Eisenstein S., Rosen B., Lau A.Y., Walter J.C., LaBaer J. (2004). Self-Assembling Protein Microarrays. Science.

[95] Milovanova T., Chatterjee S., Hawkins B.J., Hong N., Sorokina E.M., DeBolt K., Moore J.S., Madesh M., Fisher A.B. (2008). Caveolae Are an Essential Component of the Pathway for Endothelial Cell Signaling Associated with Abrupt Reduction of Shear Stress. Biochim. Biophys. Acta.

[96] Sivagnanam V., Bouhmad A., Lacharme F., Vandevyver C., Gijs M.A.M. (2008). Sandwich Immunoassay on a Microfluidic Chip Using Patterns of Electrostatically Self-assembled Streptavidin-coated Beads. Microelectron. Eng..

[97] He M., Stoevesandt O., Taussig M.J. (2008). In Situ Synthesis of Protein Arrays. Curr. Opin. Biotechnol..

[98] Cha T., Guo A., Zhu X.-Y. (2005). Enzymatic Activity on a Chip: The Critical Role of Protein Orientation. Proteomics..

[99] Talasaz A.H., Nemat-Gorgani M., Liu Y., Ståhl P., Dutton R.W., Ronaghi M., Davis R.W. (2006). Prediction of Protein Orientation upon Immobilization on Biological and non-biological Surfaces. Proc. Nat. Acad. Sci. U.S.A..

[100] Hall R.H. (2002). Biosensor Technologies for Detecting Microbiological Food Borne Hazards. Microb. Infect..

[101] Varnum S.M., Warner M.G., Dockendorff B., Anheier N.C., Lou J., Marks J.D., Smith L.A., Feldhaus M.J., Grate J.W., Bruckner-Lea C.J. (2006). Enzyme-Amplified Protein Microarray and a Fluidic Renewable Surface Fluorescence Immunoassay for Botulinum Neurotoxin Detection Using High-Affinity Recombinant Antibodies. Anal. Chim. Acta.

[102] Johnson T.J., Locascio L.E. (2002). Characterization and Optimization of Slanted Well Designs for Microfluidic Mixing under Electroosmotic Flow. Lab Chip.

[103] Chen H., Zheng Y., Jiang J.-H., Wu H.L., Shen G.L., Yu R.Q. (2008). An Ultrasensitive Chemiluminescence Biosensor for Cholera Toxin Based on Ganglioside-functionalized Supported Lipid Membrane and Liposome. Biosens. Bioelectron..

[104] Viswanathan S., Wu L.-C., Huang M.R., Ho J.A. (2006). Electrochemical Immunosensor for Cholera Toxin Using Liposomes and Poly(3,4-ethylenedioxythiophene)-coated Carbon Nanotubes. Anal. Chem..

[105] Quinn J., Patel P., Fitzpatrick B., Manning B., Dillon P., Daly S., O'Kennedy R., Alcocer M., Lee H., Morgan M., Lang K. (1999). The Use of Regenerable, Affinity Ligand-based Surfaces for Immunosensor Applications. Biosens. Bioelectron..

[106] Mayes A.G., Whitcombe M.J. (2005). Synthetic Strategies for the Generation of Molecularly Imprinted Organic Polymers. Adv. Drug Deliv. Rev..

[107] Rydholm S., Frisk T., Kowalewski J.M., Andersson Svahn H., Stemme G., Brismar H. (2008). Microfluidic Devices for Studies of Primary Cilium Mediated Cellular Response to Dynamic Flow Conditions. Biomed. Microdevices.

[108] Tan H.Y., Loke W.K., Tan Y.T., Nguyen N.T. (2008). A Lab-on-a-Chip for Detection of Nerve Agent Sarin in Blood. Lab Chip.

[109] Le Nel A., Minc N., Smadja C., Slovakova M., Bilkova Z., Peyrin J.M., Viovy J.L., Taverna M. (2008). Controlled Proteolysis of Normal and Pathological Prion Protein in a Microfluidic Chip. Lab Chip.

[110] Ellington A.D., Szostak J.W. (1990). In Vitro Selection of RNA Molecules that Bind Specific Ligands. Nature.

[111] Tuerk C., Gold L. (1990). Systematic Evolution of Ligands by Exponential Enrichment: RNA Ligands to Bacteriophage T4 DNA Polymerase. Science.

[112] Torres-Chavolla E., Alocilja E.C. (2008). Aptasensors for Detection of Microbial and Viral Pathogens. Biosens. Bioelectron..

[113] So H.-M., Won K., Kim Y.H., Kim B.K., Ryu B.H., Na P.S., Kim H., Lee J.O. (2005). Single-walled Carbon Nanotube Biosensors Using Aptamers as Molecular Recognition Elements. J. Am. Chem. Soc..

[114] Klostranec J.M., Xiang Q., Farcas G.A., Lee J.A., Rhee A., Lafferty E.I., Perrault S.D., Kain K.C., Chan W.C. (2007). Convergence of Quantum Dot Barcodes with Microfluidics and Signal Processing for Multiplexed High-Throughput Infectious Disease Diagnostics. Nano Lett..

[115] Blicharz T.M., Siqueira W.L., Helmerhorst E.J., Oppenheim F.G., Wexler P.J., Little F.F., Walt D.R. (2009). Fiber-Optic Microsphere-Based Antibody Array for the Analysis of Inflammatory Cytokines in Saliva. Anal. Chem..

[116] Lucas L.J., Chesler J.N., Yoon J.-Y. (2007). Lab-on-a-Chip Immunoassay for Multiple Antibodies Using Microsphere Light Scattering and Quantum Dot Emission. Lab Chip.

[117] Bunyakul N., Edwards K.A., Promptmas C., Baeumner A.J. (2009). Cholera Toxin Subunit B Detection in Microfluidic Devices. Anal. Bioanal.Chem..

[118] Delehanty J.B., Ligler F.S. (2002). A Microarray Immunoassay for Simultaneous Detection of Proteins and Bacteria. Anal. Chem..

[119] Pal S., Alocilja E.C., Downes F.P. (2007). Nanowire Labeled Direct-charge Transfer Biosensor for Detecting *Bacillus* Species. Biosens. Bioelectron..

[120] Diercks A.H., Ozinsky A., Hansen C.L., Spotts J.M., Rodriguez D.J., Aderem A. (2009). A Microfluidic Device for Multiplexed Protein Detection in Nano-liter Volumes. Anal. Biochem..

[121] Huh Y.S., Park T.J., Lee E.Z., Hong W.H., Lee S.Y. (2008). Development of a Fully Integrated Microfluidic System for Sensing Infectious Viral Disease. Electrophoresis..

[122] Patolsky F., Zheng G., Hayden O., Lakadamyali M., Zhuang X., Lieber C.M. (2004). Electrical Detection of Single Viruses. Proc. Nat. Acad. Sci. U.S.A..

[123] Ymeti A., Subramaniam V., Beumer T.A., Kanger J.S. (2007). An Ultrasensitive Young Interferometer Handheld Sensor for Rapid Virus Detection. Expert Rev. Med. Devices..

[124] Kirschbaum M., Jaeger M.S., Schenkel T., Breinig T., Meyerhans A., Duschl C. (2008). T Cell Activation on a Single-cell Level in Dielectrophoresis-based Microfluidic Devices. J. Chromatogr. A..

[125] Lee J., Jang J., Akin D., Savran C.A., Bashir R. (2008). Real-time Detection of Airborne Viruses on a Mass-sensitive Device. Appl. Phys. Lett..

[126] McClain M.A., Culbertson C.T., Jacobson S.C., Ramsey J.M. (2001). Flow Cytometry of *Escherichia coli* on Mirofluidic Devices. Anal. Chem..

[127] Ateya D.A., Sachs R., Besch S., Gottlieb P., Hua S.Z. (2005). Volume Cytometry: Microfluidic Sensor for High-throughput Screening in Real-time. Anal. Chem..

[128] Shelby J.P., White J., Ganesan K., Rathod P.K., Chiu D.T. (2003). A Microfluidic Model for Single-cell Capillary Obstruction by Plasmodium Falciparum-infected Erythrocytes. Proc. Nat. Acad. Sci. U.S.A..

[129] Yacoub-George E., Hell W., Meixner L., Wenninger R., Bock K., Lindner P., Wolf H., Kloth T., Feller K.A. (2007). Automated 10-channel Capillary Chip Immunodetector for Biological Agents Detection. Biosens. Bioelectron..

[130] Huh D., Gu W., Kamotani Y., Grotberg J.B., Takayama S. (2005). Microfluidics for Flow Cytometric Analysis of Cells and Particles. Physiol. Meas..

[131] Doh I., Cho Y.-H. (2005). A Continuous Cell Separation Chip Using Hydrodynamic Dielectrophoresis (DEP) Process. Sens. Actuat. B.

[132] Suehiro J., Hamada R., Noutomi D., Shutou M., Hara M. (2003). Selective Detection of Viable Bacteria Using Dielectrophoretic Impedance Measurement Method. J. Electrostat..

[133] Suehiro J., Ohtsubo A., Hatano T., Hara M. (2006). Selective Detection of Bacteria by a Dielectrophoretic Impedance Measurement Method Using an Anitbody-immobilized Electrode Chip. Sens. Actuat. B.

[134] Balagadde F.K., You L.C., Hansen C.L., Arnold F.H., Quake S.R. (2005). Long-term Monitoring of Bacteria Undergoing Programmed Population Control in a Microchemostat. Science.

[135] Futai N., Gu W., Song J.W., Takayama S. (2006). Handheld Recirculation System and Customized Media for Microfluidic Cell Culture. Lab Chip.

[136] Ertl P., Robello E., Battaglini F., Mikkelsen S.R. (2000). Rapid Antibiotic Susceptibility Testing via Electrochemical Measurement of Ferricyanide Reduction by *Escherichia coli* and Clostridium sporogenes. Anal. Chem..

[137] Ertl P., Mikkelsen S.R. (2001). Electrochemical Biosensor Array for the Identification of Microorganisms Based on Lectin-lipopolysaccharide Recognition. Anal. Chem..

[138] Peterfi Z., Kustos I., Kilar R., Kocsis B. (2007). Microfluidic Chip Analysis of Outer Membrane Proteins Responsible for Serological Cross-reaction Between Three Gram-negative Bacteria: Proteus morganii O34, *Escherichia coli* O111 and Salmonella Adelaide O35. J. Chromatogr. A..

[139] Ekins R.P. (1999). Immunoassay, DNA Analysis and Other Ligand Binding Assay Techniques: From Electrophrograms to Multiplexed, Ultrasensitive Microarrays on a Chip. J. Chem. Educ..

[140] Minerick A.R. (2008). The Rapidly Growing Field of Micro and Nanotechnology to Measure Living Cells. AICHE J..

[141] Leonard P., Hearty S., Quinn J., O'Kennedy R. (2004). A Generic Approach for the Detection of Whole Listeria Monocytogenes Cells in Contaminated Samples Using Surface Plasmon Resonance. Biosens. Bioelectron..

[142] Gomez R., Bashir R., Bhunia A.K. (2002). Microscale Electronic Detection of Bacterial Metabolism. Sens. Actuat. B.

[143] Muhammead-Tahir Z., Alocilja E.C. (2003). Fabrication of a Disposable Biosensor for *Escherichia coli* O157:H7 Detection. IEEE Sens. J..

[144] Hawkes J.J., Long J.M., Coakley W.T., McDonnell M.B. (2004). Ultrasonic Deposition of Cells on a Surface. Biosens. Bioelectron..

[145] Verporte E. (2003). Beads and Chips: New Recipes of Analyses. Lab Chip.

[146] Masafumi I., Nobuyasu Y., Katsuji T., Masao N. (2006). Rapid and Simple Detection of Food Poisoning Bacteria by Bead Assay with a Microfluidic Chip-based System. J. Microbiol. Methods.

[147] Floriano P.N., Christoduolides N., Romanovicz D., Bernard B., Simmons G.W., Cavell M., McDevitt T.T. (2005). Membrane-based On-line Optical Analysis System for Rapid Detection of Bacteria and Spores. Biosens. Bioelectron..

